# RESTORE ME: a RCT of oxaloacetate for improving fatigue in patients with myalgic encephalomyelitis/chronic fatigue syndrome

**DOI:** 10.3389/fneur.2024.1483876

**Published:** 2024-11-27

**Authors:** Alan Cash, Suzanne D. Vernon, Candace Rond, Lucinda Bateman, Saeed Abbaszadeh, Jennifer Bell, Brayden Yellman, David L. Kaufman

**Affiliations:** ^1^Terra Biological LLC, San Diego, CA, United States; ^2^Bateman Horne Center, Salt Lake City, UT, United States; ^3^Center for Complex Diseases, Seattle, WA, United States

**Keywords:** chronic fatigue syndrome, ME/CFS, oxaloacetate, fatigue, fatigue treatment, ME/CFS treatment, chronic fatigue treatment, clinical trial ME/CFS

## Abstract

**Background:**

The energy metabolite oxaloacetate is significantly lower in the blood plasma of ME/CFS subjects. A previous open-label trial with oxaloacetate supplementation demonstrated a significant reduction in myalgic encephalomyelitis/chronic fatigue syndrome (ME/CFS)-related fatigue.

**Methods:**

In this follow-up trial, 82 ME/CFS subjects were enrolled in a 3-month randomized, double-blinded, controlled study, receiving either 2,000 mg of oxaloacetate or control per day. The primary endpoints were safety and reduction in fatigue from baseline. Secondary and exploratory endpoints included functional capacity and general health status.

**Results:**

Anhydrous enol-oxaloacetate (oxaloacetate) was well tolerated at the tested doses. Oxaloacetate significantly reduced fatigue by more than 25% from baseline, while the control group showed a non-significant reduction of approximately 10%. Intergroup analysis showed a significant decrease in fatigue levels in the oxaloacetate group (*p* = 0.0039) with no notable change in the control group. A greater proportion of subjects in the oxaloacetate group achieved a reduction in fatigue greater than 25% compared to the control group (*p* < 0.05). Additionally, 40.5% of the oxaloacetate group were classified as “enhanced responders,” with an average fatigue reduction of 63%. Both physical and mental fatigue improved with oxaloacetate supplementation.

**Conclusion:**

Oxaloacetate is well tolerated and effectively helps reduce fatigue in ME/CFS patients.

**Clinical trial registration:**

https://clinicaltrials.gov/study/NCT05273372.

## Introduction

The Centers for Disease Control and Prevention (CDC) estimates that up to 3.3 million Americans suffer from Myalgic Encephalomyelitis/Chronic Fatigue Syndrome (ME/CFS) (1). ME/CFS commonly occurs after viral infections or other acutely stressful events, impacting women more frequently than men (2).

ME/CFS patients exhibit various metabolic changes, including the Warburg Effect (3) (production of energy through fermentation in the cytoplasm rather than in the mitochondria), a decrease in the NAD+/NADH ratio (4); activation of the NF-kB inflammation pathway (5); mitochondrial malfunction (6); reduced activation of AMPK (7); and possible increased production of reactive oxygen species (ROS) (8). Previous research has shown that oxaloacetate supplementation may help correct some of these energy metabolism abnormalities (9).

Oxaloacetate is a critical metabolite in the production of energy within the mitochondria through the Krebs Cycle. The Krebs cycle is a series of chemical reactions that are used to release stored energy derived from carbohydrates/sugars, fats, and proteins in the body. Normally, up to 96% of cellular energy produced involves the Krebs cycle—but in ME/CFS patients, energy metabolism is disrupted, and energy is also produced via fermentation in the cytoplasm outside of the mitochondria (3). This disrupted energy production is described as the “Warburg Effect,” a shift in ATP energy production from the Krebs Cycle and oxidative phosphorylation in the mitochondria to lactate production through glycolysis in the cell’s cytoplasm. Recently, it was discovered that oxaloacetate acts as a signaling molecule in the cytoplasm to control the Warburg Effect (10, 11). Oxaloacetate is also critical in other aspects of metabolism, including gluconeogenesis, fatty acid synthesis, amino acid synthesis, the glyoxylate cycle, the urea cycle, and the lactic acid cycle.

In mouse soleus muscle studies, acute oxaloacetate supplementation significantly enhanced fatigue resistance (12). Metabolomic analysis of the plasma of patients with ME/CFS compared to normal controls showed a significant reduction of oxaloacetate (13).

Treatment with anhydrous enol-oxaloacetate (AEO) in a 6-week, 76-patient proof-of-concept open-label trial showed significant reductions in fatigue in ME/CFS patients compared to baseline values and historical controls (9). This study is a follow-up to that trial, being a 3-month, randomized, controlled clinical trial in ME/CFS patients. Our objective was to evaluate the safety and efficacy of oxaloacetate in reducing fatigue in patients with ME/CFS.

## Materials and methods

### Study design

**Figure 1:**
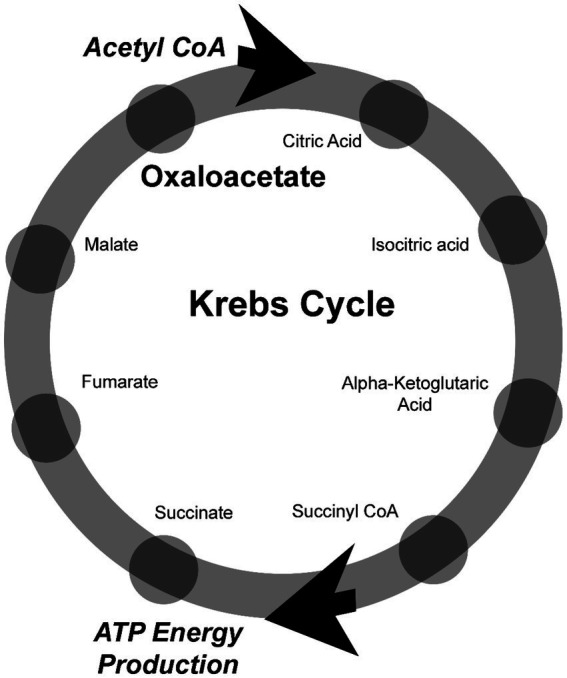
Oxaloacetate is a key part of the Krebs cycle in the mitochondria.

**Figure 2:**
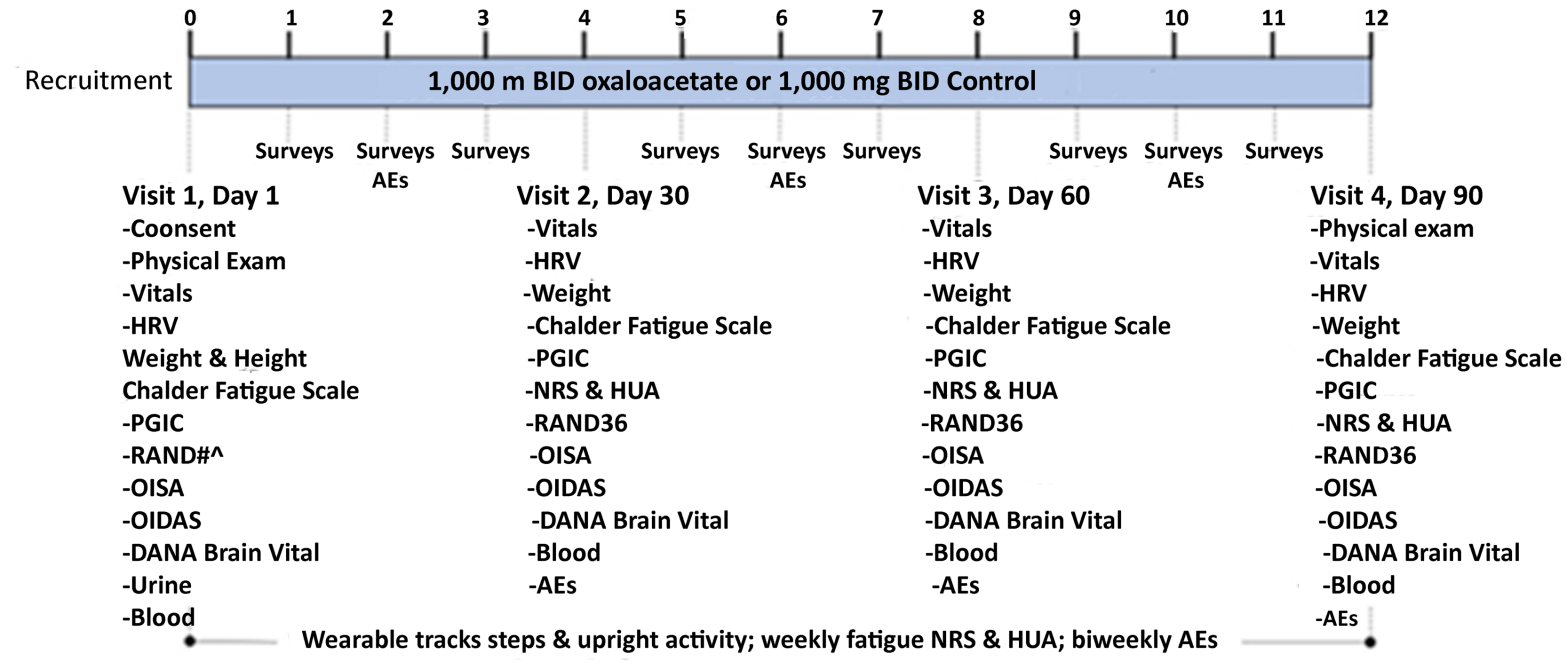
RESTORE ME schema.

RESTORE ME was a randomized, controlled, double-blinded clinical trial of an oral dose of 1,000 mg oxaloacetate or 1,000 mg rice flour, the control substance, taken twice daily (Figure 1). The trial was conducted at the Bateman Horne Center (BHC) in accordance with Good Clinical Practice and the Declaration of Helsinki and approved by the Institute of Regenerative and Cellular Medicine Institutional Review Board. This trial was registered at ClinicalTrials.gov (NCT05273372). All subjects provided written informed consent at enrollment (Figure 2).

There were four in-person visits for assessments, questionnaires, urine pregnancy testing (Visit 1 only), distribution, and compliance of oxaloacetate or control. Each subject was sent weekly online surveys and biweekly adverse event (AE) surveys. During each in-person visit, subjects were given a fully charged wearable device to be worn on the ankle for 7 days, and they were provided with a mailer to send the wearable back to BHC after 7 days. Blood samples were collected and stored for future biomarker discovery.

### Participants

RESTORE ME enrolled subjects between 18 and 65 years of age who had been diagnosed with ME/CFS with a stable state of illness in the preceding 3 months and self-reported upright activity between 2 and 6 h per day. Subjects had to have a negative COVID-19 test at the baseline visit. Subjects were excluded if there was an alternate medical or psychiatric illness that could explain the ME/CFS symptoms, if they had severe ME/CFS with less than 2 h of upright activity a day, or if they exhibited active or uncontrolled co-morbidities that could cause fatigue. Current treatment with the following stimulants was also excluded: methylphenidate, amphetamine-dextroamphetamine, lisdexamfetamine, modafinil, and armodafinil. Pregnant women, women who had given birth within the past 6 months, or women who were breastfeeding were ineligible. Participation in another clinical treatment trial or symptoms improving because of treatment intervention in the past 3 months were criteria for exclusion.

### Intervention

Oxaloacetate was administered in 500 mg capsules, which, upon exposure to water in the stomach, convert to both enol and keto oxaloacetate, both of which are human metabolites involved in energy production. Oxaloacetate is considered a medical food. The control was given as a 500 mg capsule of rice flour, used as a control food. Randomization was performed using maximally tolerated imbalance procedures, using an NIH/NCI clinical trial tool. The tool is “plug and play”: https://ctrandeomiazation.cancer.gov/tool/.

During the trial, a patient death occurred, which was classified as a serious adverse event. As a result, the trial was temporarily paused, and the study was unblinded to determine which group the deceased patient belonged to. It was revealed that the patient was in the control group, not the oxaloacetate group. After the pause, new patients were re-randomized using individual randomization numbers. This adjustment was implemented to ensure that if further serious events occurred, unblinding of other participants would not be necessary.

### Dosing

Subjects were provided with a 30-day supply of oxaloacetate or control capsules. Subjects were instructed to take two 500 mg capsules with breakfast and two 500 mg capsules with lunch daily for the three-month trial. As oxaloacetate is a “medical food,” the control for this trial was selected as another food, “white rice flour.” Dosing was monitored during on-site visits by collecting participant bottles and counting the capsules consumed. Any side effects from either oxaloacetate or control were recorded. This study had no stratification of groups, and a single dosage level of 2,000 mg/day of either oxaloacetate or control was provided daily.

The number of withdrawals was 5 out of 42 in the oxaloacetate group and 12 out of 40 in the placebo group. During the study, 92% of the oxaloacetate group and 97% of the control group were compliant with dosing based on pill counts.

### Outcomes

The primary endpoints were safety and the change in fatigue as assessed with a Patient Reported Outcome Measure (PROM), the Chalder Fatigue Scale, from Visit 1 (the baseline) to Visit 4 (the end of the 3-month study). The Chalder Fatigue Scale is an 11-item questionnaire designed to assess physical and mental fatigue (14).

### Statistical analysis

Data were summarized as means and standard deviations, and standard error and confidence intervals were calculated. Changes were summarized as effect sizes, normalized to a 0–100% scale, wherein 100% is the highest value that can be measured with the survey instrument. Normalization of the points to a 100% scale is more understandable for the ME/CFS patients who read this study. Therefore, the values were reported on a 100% scale.

Significance in Figure 4A was calculated using student’s t-test scores in Excel by comparison to baseline reductions using paired data and to the control group using two-sample equal variance (homoscedastic). As many control group patients dropped out of the study, it skewed the starting point (*p* = 0.1), so to compare control with oxaloacetate, the individual fatigue reduction of each patient was calculated as a point reduction, and then overall point reductions were evaluated using the T-Test. The Minimal Clinically Important Difference (MCID) for the binomial Chalder Fatigue Score has been established as between 1.4 and 4 points in Long COVID, a similar fatigue condition (15). In this study, clinical significance was measured by reductions of 3 or more points (> 25% fatigue reduction) in the Chalder fatigue score (bimodal scoring), as shown in Figure 4B, and by overall significant reductions in fatigue in all tests.

In Figure 5, McNemar’s test (16) was utilized to compare a 2×2 matrix and a categorical shift within each treatment group, as opposed to no shift in values—in effect, looking for changes in symmetry within each group. A low *p*-value indicates a significant shift in values, as was observed in the oxaloacetate group. The control group did not show this shift.

In Table 6, Prognostic values were calculated as an exploratory stepwise linear regression model comparing the change in the Chalder Fatigue Score at day 90 and demographic information. Due to the inherent uncertainty of prognostic examinations, a *p*-value of 0.15 was used as a cut-off, rather than the standard 0.05.

## Results

**Figure 3:**
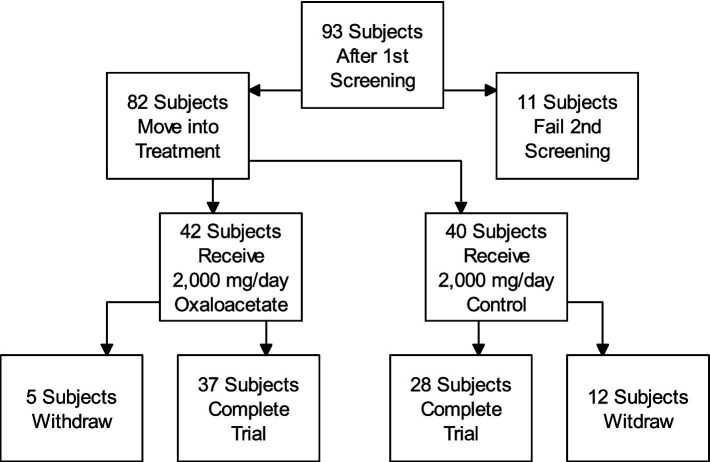
Participant flow.

Participant flow for this trial is shown in the attached diagram (Figure 3).

The clinical trial was a double-blinded, randomized, controlled study for using 2,000 mg/day oxaloacetate to treat fatigue in 82 ME/CFS subjects (oxaloacetate group *N* = 42, control group *N* = 40).

### Baseline demographics and characteristics

In total, 80% of the subjects in the oxaloacetate group were female individuals, compared to 81% in the control group. Moreover, 97% of the oxaloacetate group identified as white, whereas 93% identified as white in the control group. A total of 57% of the oxaloacetate group had an ME/CFS diagnosis for more than 5 years, compared to 42.5% of the control group (Table 1).

**Table 1:** Demographics and characteristics.

Characteristic	Oxaloacetate (*N* = 42)	Control (*N* = 40)
Age (years)		
Mean (SD)	44.2 (12.63)	45.8 (11.71)
Median (Q1, Q3)	43.5 (35.0, 56.0)	45.0 (35.5, 55.0)
Min, Max	20, 65	27, 64
Sex		
Female	34 (81.0%)	32 (80.0%)
Male	8 (19.0%)	8 (20.0%)
Ethnicity		
Hispanic or Latino	4 (9.5%)	1 (2.5%)
Not Hispanic or Latino	38 (90.5%)	36 (90.0%)
Unknown	0	1 (2.5%)
Not reported	0	1 (2.5%)
Missing	0	1 (2.5%)
Race		
White	39 (92.9%)	39 (97.5%)
Multiple	3 (7.1%)	0
Missing	0	1 (2.5%)
Duration of ME/CFS		
3 to 6 months	2 (4.8%)	2 (5.0%)
6 months to 1 year	4 (9.5%)	4 (10.0%)
1 to 2 years	5 (11.9%)	5 (12.5%)
2 to 3 years	3 (7.1%)	8 (20.0%)
3 to 4 years	3 (7.1%)	1 (2.5%)
4 to 5 years	1 (2.4%)	2 (5.0%)
5 to 6 years	6 (14.3%)	3 (7.5%)
6 to 7 years	1 (2.4%)	5 (12.5%)
7 to 8 years	2 (4.8%)	1 (2.5%)
8 to 9 years	2 (4.8%)	1 (2.5%)
9 to 10 years	2 (4.8%)	0
> 10 years	11 (26.2%)	7 (17.5%)
Missing	0	1 (2.5%)
Marital status		
Married or living with a partner	26 (61.9%)	25 (62.5%)
Separated	0	1 (1.2%)
Divorced	5 (11.9%)	7 (17.5%)
Widowed	0	1 (2.5%)
Never married	11 (26.2%)	5 (12.5)
Missing	0	1 (2.5%)
Education level		
High school graduate	5 (11.9%)	1 (2.5%)
GED or equivalent	1 (2.4%)	0
Some college, no degree	6 (14.3%)	8 (20.0%)
Associate degree: occupational	4 (9.5%)	4 (10.0%)
Associate degree: academic	2 (4.8%)	4 (10.0%)
Bachelor’s degree	11 (26.2%)	13 (32.5%)
Master’s degree	8 (19.0%)	6 (15.0%)
Professional school degree	2 (4.8%)	1 (2.5%)
Doctoral degree	0	1 (2.5%)
Refused	3 (7.1%)	1 (2.5%)
Missing	0	1 (2.5%)
Number of children		
0	15 (35.7%)	11 (27.5%)
1	4 (9.5%)	3 (7.5%)
2	9 (21.4%)	10 (25.0%)
3	8 (19.0%)	8 (20.0%)
4 or more	4 (9.5%)	6 (15.0%)
Missing	2 (4.8 T)	2 (5.0%)
Working status		
Working now	15 (35.7%)	14 (35.0%)
Only temporarily laid off	1 (2.4%)	1 (2.5%)
Looking for work, unemployed	2 (4.8%)	2 (5.0%)
Retired	2 (4.8%)	1 (2.5%)
Disabled	13 (31.0%)	15 (37.5%)
Keeping house	5 (11.9%)	4 (10.0%)
Student	1 (2.4%)	0
Other	3 (7.1%)	2 (2.5%)
Missing	0	1 (2.5%)

### Safety

Our primary endpoints in the trial were safety and the reduction of fatigue. There were no remarkable changes in vital signs between the two groups. There were no serious treatment-emergent adverse events (TEAE) in the oxaloacetate group. TEAE is defined as any event reported on or after the registration date. There were three serious TEAEs in the control group (including one death). The most common possibly related non-serious TEAEs in the oxaloacetate group (greater than 5%) were headache and nausea (3 each). The control group had many similar non-serious TEAEs but experienced six severe cases. The oxaloacetate group had two cases of non-serious TEAEs that were severe, with nausea and abdominal pain in one patient. Overall, oxaloacetate was well tolerated by this participant group (Table 3).

**Table 2:** Non-serious treatment-emergent adverse events (TEAE).

Related treatment-emergent adverse events Reported term	Oxaloacetate (*N* = 42)	Control (*N* = 40)
Headache	3 (7.1%)	0
Nausea	3 (7.1%)	1 (2.5%)
Diarrhea	1 (2.4%)	1 (2.5%)
Abdominal cramping	2 (4.8%)	0
Abdominal pain	2 (4.8%)	0
Change in bowel habits	1 (2.4%)	1 (2.5%)
Chest pain	0	1 (2.5%)
Migraine	0	1 (2.5%)
Urinary tract infection	0	1 (2.5%)
Anorexia	1 (2.4%)	0
Cervicogenic headache	1 (2.4%)	0
Dry nostril	1 (2.4%)	0
Jackhammer esophagus	0	1 (2.5%)
Hot flashes	1 (2.4%)	0
Intermittent chest pressure	1 (2.4%)	0
Lower abdominal pain	1 (2.4%)	0
Myalgias of rhomboids along the spine	1 (2.4%)	0
Numbness of left leg	0	1 (2.5%)
Numbness of the face	0	1 (2.5%)
Numbness of the left arm	0	1 (2.5%)
Pain in the left side of the jaw	0	1 (2.5%)
Pruritic left arm	0	1 (2.5%)
Sinus infection	0	1 (2.5%)
Sore muscles	1 (2.4%)	0
Stomach upset	1 (2.4%)	0
Thoughts of self-harm	0	1 (2.5%)
Vomit	0	1 (2.5%)
Word finding difficulty	1 (2.4%)	0
Worsening of hypertension	0	1 (2.5%)

**Table 3:** Total TEAE by severity.

	Oxaloacetate (*N* = 42)	Control (*N* = 40)
Total number of TEAEs	41	40
Number (%) of participants reporting at least one		
TEAE	22 (52.4%)	16 (40.0%)
Serious TEAE	0	3 (7.5%)
Related TEAE	12 (28.6%)	7 (17.5%)
Maximum severity		
Mild	10 (23.8%)	5 (12.5%)
Moderate	11 (26.2%)	5 (12.5%)
Severe	2 (4.8%)	6 (15.0%)

The one participant in the oxaloacetate group who experienced severe nausea and abdominal pain dropped due to this TEAE. In contrast, all other oxaloacetate participants dropped for reasons other than TEAEs.

### Fatigue

**Figure 4:**
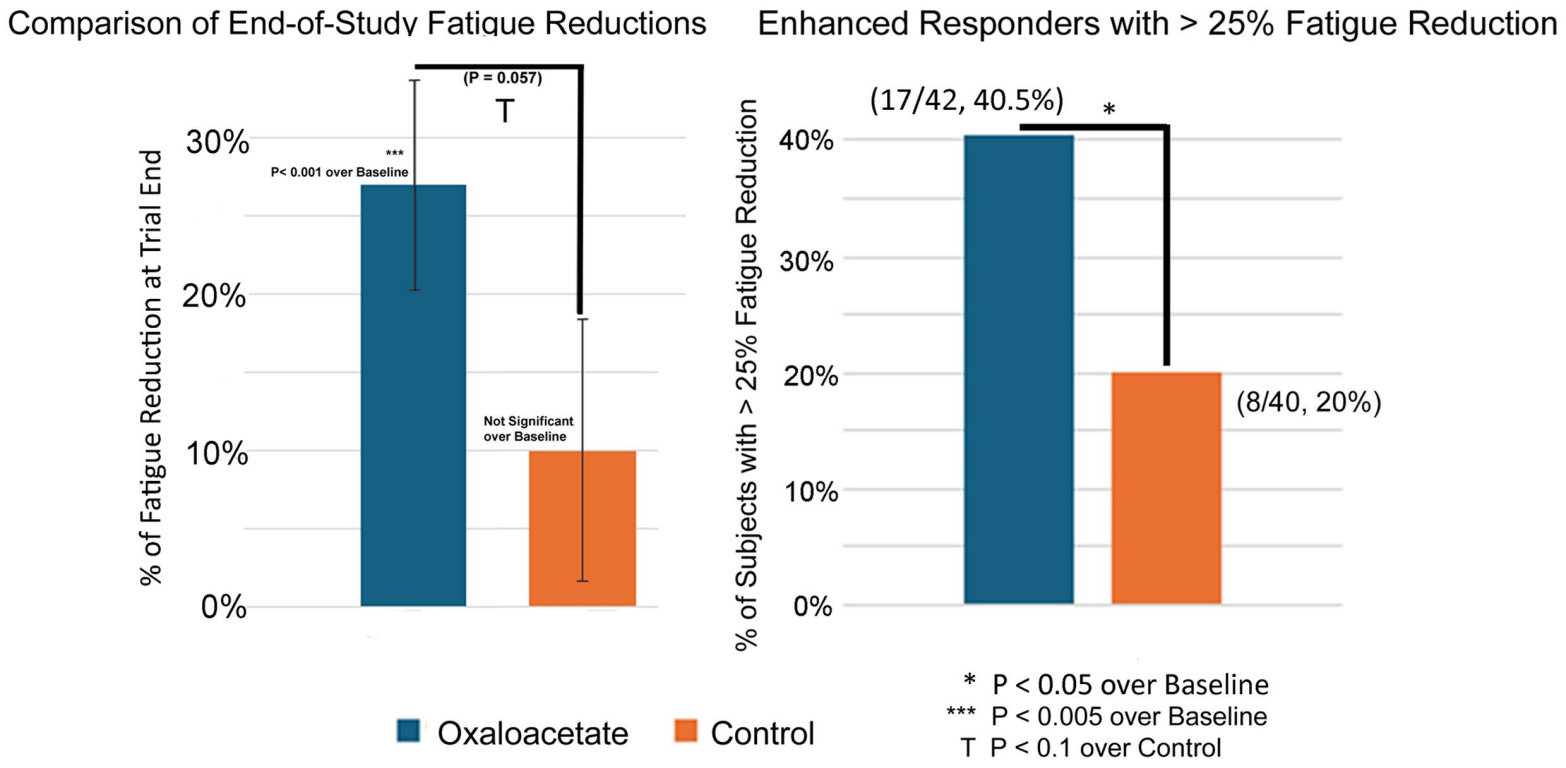
**(A,B)** 3-month (end-of-study) reductions in fatigue and percentage of patients with >25% reduction in fatigue.

The Chalder Fatigue Score was designated as this trial’s primary measurement of fatigue reduction efficacy. The oxaloacetate group saw significant average reductions in fatigue from baseline of 27%, whereas the control group reduction was not significant (Figure 4A). The oxaloacetate group trended toward significance over the control at the study end (*p* = 0.057).

The oxaloacetate group contained a subgroup of “*enhanced responders*,” defined as patients with greater than 25% fatigue reduction. The number of enhanced responders was significant over the control (40.5% oxaloacetate group vs. 20% control group, Figure 4B) and *demonstrated a 63% reduction in fatigue*.

Intergroup analysis of both the complete set of all participants (including dropouts) and participants who made it through the entire 3-month trial showed a significant shift to lower fatigue levels in the oxaloacetate group but not in the control group (*p* = 0.0039 oxaloacetate), (*p* = 0.4531 control group “completed subjects”) (*p* = 0.1094 control group “All Subjects”).

**Figure 5:**
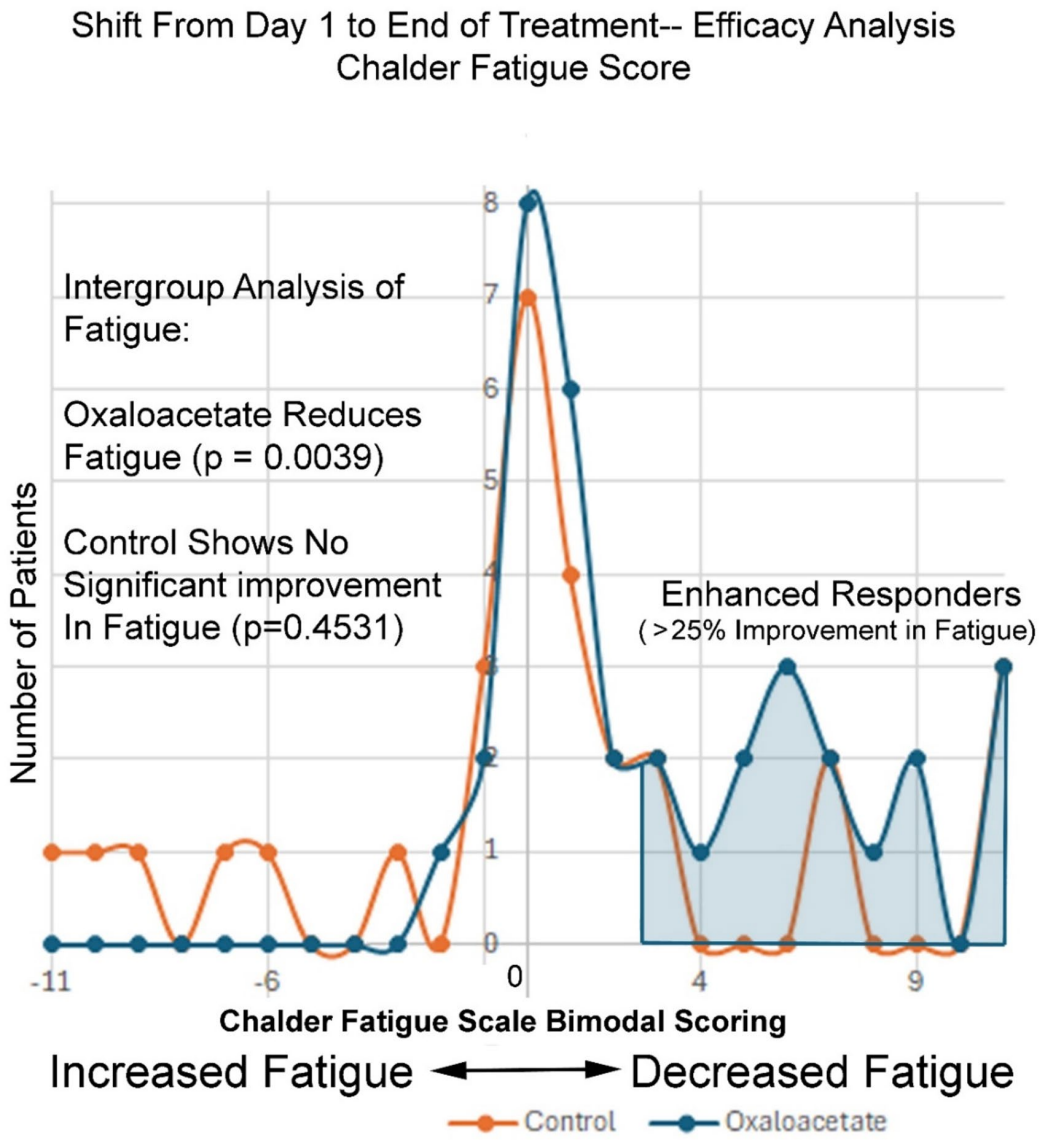
Oxaloacetate reduces fatigue levels, especially for “enhanced responders”.

Figure 5 shows the frequency of Chalder Fatigue Scores as changed from the baseline to the end of the 3-month study period. The normal distribution of fatigue change across the control group shows a bell-shaped normal distribution of fatigue values, with most values showing little to no change and outlier values showing both negative and positive changes in fatigue at the end of the study. The negative 11 to positive 11-point scale records the change in Chalder Fatigue Score from the baseline to the end of the study, with each division equal to one point on the Chalder bimodal scale. In contrast, the oxaloacetate group shows nearly no negative values, as the entire group’s fatigue levels were shifted to the right (decreased fatigue). On the right of Figure 5, the enhanced responder group can be observed (Table 4).

**Table 4:** Chalder fatigue score reductions.

* Indicates *p* < 0.05 over Baseline	Oxaloacetate		Improvement over baseline	Control		Improvement over baseline
	Baseline	End		Baseline	End	
Chalder fatigue score, all participants	9.1	6.6	27% ***	8.5	7.1	17% *
Chalder fatigue score, trial completed participants	9.1	6.2	32%*** T (over control)	8.1	7.0	14%
CFS, *N* of enhanced responders >25% improvement		17/42	40.5% * (over control)		8/40	20%
OAA enhanced responders % fatigue improvement			63% ***			
CFS, McNemar’s shift analysis, full set *P* value			*p* = 0.0039 ***			*P* = 0.1094
McNemar’s shift analysis, completed participants *P*			*p* = 0.0039 ***			*P* = 0.4531

An exploratory stepwise linear regression model was constructed to analyze the effect of potential demographic prognostic factors that might lead to a positive change of greater than 25% in the Chalder Fatigue Score (CFS, bimodal). Factors that improved the chances of moving to the “Enhanced Responder” group, using a significance of 0.1500 in the model, included the initial baseline value of the Chalder Fatigue Scale, duration of ME/CFS, RAND-36 Energy/Fatigue baseline value, and if the participant was seeking employment. These variables may be potential predictors of an improvement of >25% in the Chalder Fatigue Score, “enhanced responders,” by the end of this 3-month study.

Other demographic variations, including age, sex, ethnicity, race, marital status, education level, and number of children, failed to meet a predictive significance of at least 0.1500, Supporting the results of the Chalder Fatigue Scale, the RAND-36 Energy/Fatigue score was significant over baseline for the oxaloacetate group (*p* = 0.012) but not for the control group (*p* = 0.16). Fatigue reduction was also significant for oxaloacetate over control (*p* = 0.044).

## Discussion

Oxaloacetate was well tolerated at 2,000 mg/day. The most common treatment-emergent effects in this 3-month study were headache (3/42) and nausea (3/42). Anecdotal reports from other subjects taking higher levels of oxaloacetate report that if nausea occurs, it is reduced when the oxaloacetate is taken with a full meal.

In the Chalder Fatigue Scores, there was an overall 27 to 32% reduction of fatigue, which was a significant, sustained change over baseline in the oxaloacetate group in a 3-month study. The control group had a 17% reduction in fatigue, which was only significant if participants who dropped out of the study were included in the calculations. The control group failed to reach significance with subjects that completed the trial. Inter-group analysis of both the complete set of all participants (including dropouts) and participants that made it through the entire 3-month trial showed a significant shift to lower fatigue levels in the oxaloacetate group but not in the control group (*p* = 0.0039 oxaloacetate), (*p* = 0.4531 control group “completed subjects”) (*p* = 0.1094 control group “all subjects”). This group shift in fatigue toward better energy levels is observed across the entire oxaloacetate group. Taking each improvement point on the Chalder Fatigue Scale and viewing the frequency from the mean, this shift included low responders that improved a group total of 42 points above expectation (compared to the control group) and enhanced responders that improved 60 points above expectation. The shift toward increased energy levels also significantly increased the number of “enhanced responders,” defined as patients with fatigue reduction of greater than 25% (40.5% oxaloacetate group vs. 20% control group). Furthermore, 40.5% of participants in the oxaloacetate group who comprised the “enhanced responders” saw a 63% improvement in fatigue.

The Chalder Fatigue Scale is often used in ME/CFS studies and is validated for mild and moderate ME/CFS fatigue. Very severe cases have a “ceiling effect” problem, in which the fatigue levels are too high to measure with the Chalder Fatigue Scale (17).

The RAND-36 is also used in ME/CFS studies and is validated for mild and moderate ME/CFS. The oxaloacetate group had a significant 35% reduction in fatigue over baseline, while the control group was not significant in this measurement. The control group’s reduction in energy/fatigue was not significant. This second survey supports the findings using the Chalder Fatigue Scale.

Both the Chalder Fatigue Scale and the RAND-36 are patient-reported outcome measures (PROM) and are influenced by multiple sources of bias, including variations in the interpretation of the questions and response options (18). Another source of bias may have been that subjects at this single-center trial may have shared their results, but attempts to minimize contact were made. Since the enhanced responders saw an average improvement in fatigue of 63% in the Chalder Fatigue Score, it may have influenced others in the oxaloacetate and control groups, making PROMs less viable.

Table 5 discusses the potential prognostic demographic factors that may increase the probability of patients seeing a greater than 25% improvement in fatigue as measured by the Chalder Fatigue Scale by the 90-day point. These demographic factors include a positive association with increased baseline Chalder Fatigue Scores and a negative association with increased baseline RAND-36 Energy/Fatigue score, length of ME/CFS illness, and unemployment status. The significance of the bimodal CFS baseline value as a prognostic factor may not be important. Higher baseline values are more likely to improve, regardless of treatment received, because there is more room for improvement. However, this is argued against by the fact that the RAND-36 Energy/Fatigue scale shows the opposite effect, e.g., decreases in baseline values are associated with better fatigue treatment.

**Table 5:** Exploratory prognostic stepwise linear regression model to place into the enhanced responder group.

Step	Variable entered	Partial R-square	Model R-square	C (p)	*F* value	Pr > F
1	Bimodal CFS baseline value	0.1749	0.1749	2.1134	12.3	0.0009
2	Duration of ME/CFS	0.0995	0.2744	−2.8935	7.82	0.007
3	RAND-36 fatigue baseline	0.0357	0.3101	−3.4053	2.89	0.0944
4	Looking for work, unemployed	0.032	0.342	−3.6579	2.67	0.1077

A strong significant correlation (0.007) was observed between the duration of ME/CFS and the likelihood of an enhanced response to oxaloacetate treatment—the longer the duration of ME/CFS, the more likely the participant was to exhibit a stronger response. However, this could be attributed to patients with longer disease duration starting with higher fatigue levels. The negative association with unemployment is likely due to the small sample size—only two participants in the oxaloacetate group were classified as “unemployed, looking for work.” Therefore, despite the low *p*-values in the model, the prognostic factors based on patient demographics remain unclear in this study. We anticipate that the ongoing metabolomic analysis of the patients will provide more insight into prognostic factors for improvement.

There are multiple mechanisms by which oxaloacetate may be used to treat patients with ME/CFS. The identified mechanisms are listed below in Table 7 and include improvements to cellular energy functioning, immune system reset, glucose system functioning, and neurological antioxidant protection. We are currently unsure which of these multiple mechanisms may be the most effective in reducing fatigue. Blood samples collected during the study may provide valuable insights, and metabolic analysis of the samples is currently underway at multiple facilities. The improvement in fatigue levels observed in the oxaloacetate group aligns with the improvement found in the previous “proof-of-concept” clinical trial.

**Table 6:** RAND-36 scoring for oxaloacetate and control.

Rand 36 scoring	Oxaloacetate			Control		
* Indicates *p* < 0.05 over baseline T Indicates *p* < 0.1 over baseline	Baseline	Visit 4	Improvement over baseline	Baseline	Visit 4	Improvement over baseline
Rand 36 Physical functioning	20.1	20.3	1%	19.4	19.4	0%
Rand 36 Role limitations due to Physical Health	4.1	13.5	233%*	5.2	14.3	167%
Rand 36 Role limitations due to Emotions	47.7	54.1	13%	33.3	40.5	24%
Rand 36 Energy/fatigue	20.3	27.3	35%*	20.7	25.3	23%
Rand 36 Emotional wellbeing	63.1	63.9	1%	60.1	60.3	0%
Rand 36 Social functioning	32.33	33.04	−11%	39.19	34.8	2%
Rand 36 Pain	50.3	55.6	10% T	49.5	55	14%*
Rand 36 General health	32.8	35.5	8%	36.5	38.2	7%
Rand 36 Health change	47.3	50	6%	54.3	55.4	−2%

**Table 7:** Oxaloacetate metabolic changes that may improve fatigue in ME/CFS patients.

Metabolic change	Effect on ME/CFS patient	Normalization by oxaloacetate
Warburg effect	Increased lactate production	Reduction in lactate production via inhibition of lactate dehydrogenase in the cytosol
Decrease in NAD+/NADH ratio	Increase in ROS Production	Reset of NAD+/NADH ratio and quenching of ROS by antioxidant oxaloacetate
Increased NF-kB movement to the nucleus	Activation of chronic inflammation	Reset of the inflammation pathway to normal by lowering NF-kB translocation to the nucleus
Mitochondrial damage	Reduced ability to process glucose	Increased number of mitochondria to produce energy via PGC1-alpha increase
Reduced AMPK activation	Reduced cellular glucose uptake	Increase in glucose uptake via AMPK activation and more glucose fuel available for the patient
Increased neurological ROS production	Damage from free radicals	Oxaloacetate is a highly effective antioxidant.

In our previous study, we suggested that the improvements in fatigue may be due to the normalization of dysfunctional metabolic pathways. Briefly, we discussed the known metabolic changes induced by oxaloacetate that could potentially reduce fatigue.

### Abnormal energy production via increased glycolysis and increased lactate production in ME/CFS cells (the “Warburg Effect”)

Cells from individuals with ME/CFS show abnormal energy production, wherein more energy is produced within the cytoplasm via increased glycolysis and fermentation instead of being produced in the mitochondria (3). This is also known as the “Warburg Effect” and is common in cancer cell metabolism. Once considered a permanent metabolic change, in the past few years, providing oxaloacetate to the cytoplasm of the cells has been shown to reverse the Warburg effect (10, 11). This reduces lactate production in the cell, which is a common issue with ME/CFS patients. The abnormal elevation in lactate levels with exercise in ME/CFS patients is postulated to cause Post Exertional Malaise (PEM) (19).

### Cells from patients with fatigue show significantly lower redox (NAD + /NADH ratio) levels

Fatigued patients show a decrease in the NAD+/NADH levels in the cytoplasm (4). This redox imbalance can be overcome with oxaloacetate (20, 21). The increase in NAD+ levels and decrease in NADH levels are caused by the conversion of oxaloacetate to malate via the cytosolic enzyme malic dehydrogenase. The biochemical reaction has a Gibbs free energy of −29.7 kJ/mol, indicating a high probability that this reaction spontaneously occurs. Krebs measured the NAD + /NADH ratio change with supplemental oxaloacetate as a 900% increase within 2 min (22).

### Persistent NF-kB inflammation reduction

Cells from individuals with ME/CFS show increased activation of NF-kB, leading to persistently elevated levels of inflammatory proteins (5). Oxaloacetate has been shown to reduce the activation of NF-kB by up to 70% in animal models (20) by reducing the translocation of NF-kB from the cytosol to the nucleus. The reduction in chronic inflammation may decrease persistent fatigue in ME/CFS patients.

### Mitochondrial damage is prevalent in ME/CFS patients

Mitochondrial damage is a suggested mechanism of ME/CFS (6, 23). Oxaloacetate has been shown to increase mitochondrial density via the upregulation of PGC-1 alpha (20). Increasing mitochondrial density will allow the replacement of damaged mitochondria.

### AMP-activated protein kinase (AMPK) activation reduction

Failure of AMPK activation has been shown in ME/CFS patients (7). AMPK activation is critical for increasing glucose uptake into the cells during times of low energy (7, 24). Oxaloacetate increases AMPK activation via an increase in the NAD+/NADH ratio. It has been used in diabetic and Alzheimer’s trials to improve glucose uptake (25, 26). Having enough glucose for energy production reduces fatigue.

### Reactive oxygen species (ROS) reduction

ROS overproduction due to the Warburg Effect observed in ME/CFS cells can damage cellular components. Oxaloacetate is a powerful antioxidant, reducing both thiobarbituric acid and hydrogen peroxide in the brain (27, 28). Oxaloacetate also protects mitochondrial DNA from damage by agents such as Kainic acid (29). Reducing ROS production will help keep the cells operating successfully.

These six metabolic changes in ME/CFS and other fatigue patients may be the driving force of fatigue. Normalization of these metabolic changes by oxaloacetate may restore a non-fatigue state.

A summary table of metabolic issues in ME/CFS and the potential effects of oxaloacetate on these pathways is republished in Table 2 (9).

The participants in this study had mild to moderate ME/CFS and were specifically selected because changes in their fatigue level could be measured using existing validated surveys without encountering the “ceiling effect” caused by excessively high fatigue levels. Future studies may consider exploring the effect of oxaloacetate on patients with severe ME/CFS, provided that validated surveys suitable for this population are developed.

## Conclusion

This 3-month, double-blinded, randomized, controlled clinical trial showed that oxaloacetate was well tolerated in ME/CFS patients at a dosage of 2,000 mg/day (1,000 mg BID with a meal). The study showed significant and sustained reductions in fatigue from baseline, ranging between 27 and 32%, which were not observed in the control group. Intergroup analysis showed a significant reduction in fatigue levels in the oxaloacetate group, which was absent in the control group. Additionally, approximately 40% of the oxaloacetate group, termed “enhanced responders,” exhibited an average fatigue reduction of 63% from baseline, and this subgroup was significantly larger compared to the control group.

Oxaloacetate has proven to be an effective and well-tolerated medical food for reducing fatigue in individuals with mild to moderate ME/CFS. Further research is required to evaluate its potential benefits in alleviating fatigue among patients with severe ME/CFS.

## Data Availability

The datasets presented in this study can be found in online repositories. The names of the repository/repositories and accession number(s) can be found at: https://doi.org/10.7910/DVN/KBCE6H.
